# Response of aerobic anoxygenic phototrophic bacteria to limitation and availability of organic carbon

**DOI:** 10.1093/femsec/fiae090

**Published:** 2024-06-17

**Authors:** Kasia Piwosz, Cristian Villena-Alemany, Joanna Całkiewicz, Izabela Mujakić, Vít Náhlík, Jason Dean, Michal Koblížek

**Affiliations:** Department of Fisheries Oceanography and Marine Ecology, National Marine Fisheries Research Institute, 81-332 Gdynia, Poland; Laboratory of Anoxygenic Phototrophs, Institute of Microbiology of the Czech Academy of Sciences, 379 01 Třeboň, Czechia; Department of Fisheries Oceanography and Marine Ecology, National Marine Fisheries Research Institute, 81-332 Gdynia, Poland; Laboratory of Anoxygenic Phototrophs, Institute of Microbiology of the Czech Academy of Sciences, 379 01 Třeboň, Czechia; Faculty of Fisheries and Protection of Waters, South Bohemian Research Center of Aquaculture and Biodiversity of Hydrocenoses, Institute of Aquaculture and Protection of Waters, University of South Bohemia, 389 25 České Budějovice, Czechia; Laboratory of Anoxygenic Phototrophs, Institute of Microbiology of the Czech Academy of Sciences, 379 01 Třeboň, Czechia; Laboratory of Anoxygenic Phototrophs, Institute of Microbiology of the Czech Academy of Sciences, 379 01 Třeboň, Czechia; Department of Ecosystem Biology, Faculty of Science, University of South Bohemia, 370 05 České Budějovice, Czechia

**Keywords:** acetate, aerobic anoxygenic phototrophic bacteria, carbon limitation, freshwater lakes, lignin, microbial ecology

## Abstract

Aerobic anoxygenic phototrophic (AAP) bacteria are an important component of freshwater bacterioplankton. They can support their heterotrophic metabolism with energy from light, enhancing their growth efficiency. Based on results from cultures, it was hypothesized that photoheterotrophy provides an advantage under carbon limitation and facilitates access to recalcitrant or low-energy carbon sources. However, verification of these hypotheses for natural AAP communities has been lacking. Here, we conducted whole community manipulation experiments and compared the growth of AAP bacteria under carbon limited and with recalcitrant or low-energy carbon sources under dark and light (near-infrared light, λ > 800 nm) conditions to elucidate how they profit from photoheterotrophy. We found that AAP bacteria induce photoheterotrophic metabolism under carbon limitation, but they overcompete heterotrophic bacteria when carbon is available. This effect seems to be driven by physiological responses rather than changes at the community level. Interestingly, recalcitrant (lignin) or low-energy (acetate) carbon sources inhibited the growth of AAP bacteria, especially in light. This unexpected observation may have ecosystem-level consequences as lake browning continues. In general, our findings contribute to the understanding of the dynamics of AAP bacteria in pelagic environments.

## Teaser

Freshwater aerobic anoxygenic phototrophic bacteria may profit from photoheterotrophy under carbon-rich and poor conditions.

## Introduction

Photoheterotrophic bacteria are an abundant part of bacterioplankton. These organisms depend on organic matter for their growth, but they can supplement their energy requirements with light. One of the main photoheterotrophic groups in aquatic environments is aerobic anoxygenic phototrophic (AAP) bacteria, which harvest light by bacteriochlorophyll (BChl) and carotenoid molecules bound to photosynthetic complexes to produce ATP via cyclic photophosphorylation (Okamura et al. 1986, Yurkov and Beatty 1998). Photoheterotrophic metabolism in AAP bacteria is affected by growth in the light/dark cycle (contrary to constant light or dark), irradiance, presence of oxygen, and amount and type of carbon source (Yurkov and Van Gemerden 1993, Koblížek et al. 2010). BChl production in cultures is repressed by high concentrations of organic substrates (Kopejtka et al. 2021, Kuzyk et al. 2023). When resources are scarce, illuminated AAP bacteria can repurpose their usage from respiration to biomass production (Hauruseu and Koblížek 2012, Piwosz et al. 2018a, Koblížek et al. 2020). However, how the additional energy from light is utilized to provide an advantage for AAP bacteria in the environment remains unknown.

AAP bacteria were discovered in coastal marine waters (Shiba et al. 1979, 1991). Later, they were also found to be common in the open ocean (Kolber et al. 2001), where they typically represent 1–10% of total bacteria (reviewed in Koblížek 2015). Initially, it was hypothesized that the photoheterotrophy represents an advantage in nutrient-poor oceans, which seems to be correct for rhodopsin-containing photoheterotrophs, but AAP bacteria prefer more productive coastal areas (Gómez-Consarnau et al. 2019, Vrdoljak Tomaš et al. 2019).

AAP bacteria commonly contribute from <1 to >30% of total bacteria in freshwater lakes (Yurkov and Gorlenko 1990, Masin et al. 2008). AAP cells are on average larger, more active and have higher growth rates than heterotrophic bacteria (Fauteux et al. 2015, Cepáková et al. 2016, Garcia-Chaves et al. 2016), thus they contribute more to the microbial food webs than their abundance alone would indicate, both as consumers of phytoplankton-derived dissolved organic matter (Piwosz et al. 2020, 2022) and as a food source for bacterivores (Ruiz-González et al. 2020). Abundances of lacustrine AAP bacteria correlate positively with total phosphorous, chlorophyll-*a* concentrations and dissolved organic carbon (Masin et al. 2012, Čuperová et al. 2013, Kolářová et al. 2019). Moreover, their seasonal peaks often closely follow phytoplankton blooms (Lew et al. 2015, Kolářová et al. 2019, Villena-Alemany et al. 2024). Taken together, it seems that freshwater AAPs, similar to their marine counterparts, also prefer more productive waters, contradicting the hypothesis of better survival in the oligotrophic environment. Furthermore, it was also suggested that photoheterotrophy may help AAP bacteria to access low energetic and recalcitrant carbon sources (Salka et al. 2014, Koblížek 2015), but this has not been experimentally tested yet in natural communities.

Here, we conducted the whole microbial community manipulation experiment from a freshwater lake to test hypotheses that (i) the additional energy from light provides AAP bacteria with an advantage under carbon-limiting conditions; and that (ii) it facilitates them to access low energetic or recalcitrant carbon sources. The incubations were performed in the dark and in the infrared (IR) light (λ>800 nm), which selectively excited the IR *in vivo* absorption band of bacteriochlorophyll in AAPs or, in anaerobic conditions, purple non-sulfur bacteria (Kasalický et al. 2018, Kopejtka et al. 2020). Microorganisms that lack BChl, such as oxygenic photoautotrophs, rhodopsin-containing bacteria and chemoorganotrophs, perceived both conditions as dark. We followed the bulk growth rates of heterotrophic and AAP bacteria in dark vs IR light in the conditions of (i) organic carbon limitation; (ii) low energetic or recalcitrant organic carbon source; and (iii) natural organic carbon availability (control). We expected that in the conditions that favor photoheterotrophic metabolism, AAP bacteria would grow faster in the IR light and, as a result, they would increase their contribution to the total bacterial abundance. Moreover, to account for the metabolic differences between different AAP phylotypes, we also followed the changes in their community.

## Materials and methods

### Setting up the experiments

We conducted two experiments in June and October 2018. Water was collected from 0.5 m depth of the meso-oligotrophic freshwater lake Cep (48°55′29.7“N, 14°53′12.5″E) using a Ruttner Water Sampler (model 11.003KC Denmark AS) on 20 June 2018 and 1 October 2018. It was transported to the laboratory within 30 min in a closed plastic container, which was prerinsed three times with the sampled water and stored in a cool box.

**Figure 1.**
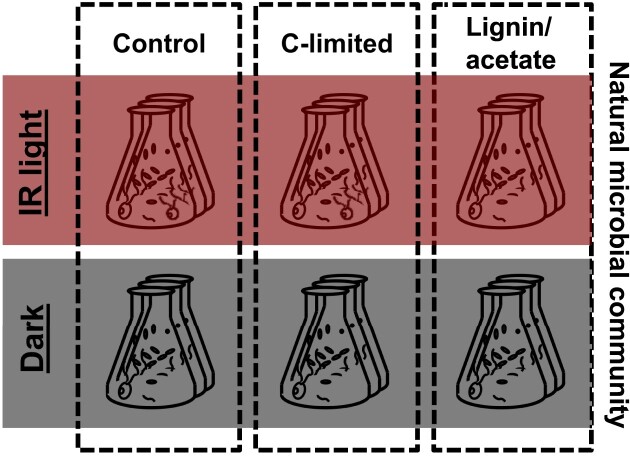
Experimental design. Natural microbial communities were diluted 1 : 4 with sterile filtered lake water (control), sterile inorganic medium (C-limited) or sterile inorganic medium containing lignin (in June) or acetate (in October) as carbon source, and incubated in the dark or under infrared illumination (IR light). All treatments were performed in triplicate.

Two different treatments were prepared: carbon limitation (C-limited), a recalcitrant organic carbon source (lignin) in June, and a low-energy organic carbon source (acetate) in October (Fig. 1). Lignin is an important component of plant biomass, and thus of the organic matter of terrestrial origin, and it can be degraded by genera that also contain AAP bacteria (Bugg et al. 2011). However, as we did not observe much growth, we decided to use acetate in October, as this compound is well known to be utilized by AAP bacteria (Yurkov and Van Gemerden 1993), although with lower efficiency than glucose (Hauruseu and Koblížek 2012).

C-limited treatment was prepared by diluting the untreated water from the lake at a 1 : 4 ratio with an 1 : 1500× diluted unamended sterile inorganic basal (1 : 1500×IBM) medium (Hahn et al. 2003). For the lignin/acetate treatments, the untreated water from the lake was diluted 1 : 4 with a sterile 1 : 1500×IBM medium containing 2.5 mg L^−1^ of dissolved lignin (in the June experiment) or 3.0 mg L^−1^ of acetate (in October). The media were prepared during the week before the experiment. They were filtered through a 0.2 µm filter and autoclaved. As a control, we used the untreated water from the lake diluted 1 : 4 with sterile filtered lake water that was collected 3 days before the experiment. It was sequentially prefiltered through a 20 µm plankton net, 0.2 µm filter and 1 L Stericup® Filter Units with a membrane pore size of 0.1 µm (Millipore, Merck) and kept in the dark at 4°C until the experiment. The dilution allowed for an increase in the carbon availability for bacteria and a reduction of the grazing pressure from protistan grazers. The concentrations of dissolved organic carbon (DOC) were measured in the bulk volume before autoclaving, according to standard methods in limnology at the Institute of Hydrobiology, Biology Centre, Czech Academy of Sciences (Shabarova et al. 2021). In June, the DOC concentration was 4.36 mg C L^−1^ in the control treatment, 1.33 mg C L^−1^ in the C-limited treatment and 1.88 mg C L^−1^ in the lignin treatment. In October, it was 4.28 mg C L^−1^ in the control treatment, 1.21 mg C L^−1^ in the C-limited treatment and 1.82 mg C L^−1^ in the acetate treatment.

Each treatment was divided into six 2 L portions that were incubated in the dark or IR light at *in situ* temperature (21°C in June and 16°C in October) in triplicate. To ensure aerobic conditions, the containers had wide openings that were covered only with sterile tin foil, which prevented contamination and allowed for gas exchange. The incubation time was 56 h (with samples taken every 12 h) in June, and 96 h (with samples taken every 24 h) in October. The reason for this difference was that at higher temperatures the initial response of bacteria to the treatment (which was our main interest here) is fast, and we wanted to avoid long incubations, during which the unanticipated processes could confound our interpretations.

### Bacterial and AAP abundance

Samples of 50 ml were fixed with buffered, sterile-filtered paraformaldehyde (Penta, Prague, Czechia) to a final concentration of 1% and 0.5 ml was filtered onto white polycarbonate filters (pore size 0.2 µm; Nucleopore, Whatman, UK). Cells were stained with 4′,6-diamidino-2-phenylindole at a concentration of 1 mg L^−1^ (Coleman 1980). Total and AAP bacterial abundances were determined using an epifluorescence Zeiss Axio Imager.D2 microscope (Cottrell et al. 2006, Villena-Alemany et al. 2023). The abundance of heterotrophic bacteria was calculated as the difference between the total bacteria and AAP bacteria.

### DNA extraction, amplicon preparation and sequencing

About 350 ml of water was filtered onto sterile 0.2 µm Nucleopore Track-Etch Membrane filters (Whatman; Maidstone, UK). Filters were placed inside sterile cryogenic vials containing 0.55 g of sterile zirconium beads, flash-frozen in liquid nitrogen and stored at −80°C until DNA extraction (<6 months). Total nucleic acids were chemically extracted according to Griffiths et al. (2000) with modifications (Nercessian et al. 2005). Extracted DNA was re-suspended in 35 µl of DNase and RNase-free water (MP Biomedicals, Solon, OH, USA) and stored at −20°C. The concentration and quality of the extracts were checked using a NanoDrop (ThermoFisher Scientific). A pure culture of *Dinoroseobacter shibae* was used as a control for cross-contamination between the samples.

Amplicons for the *puf*M gene (a marker gene for AAP bacteria) were prepared using *puf*M UniF and *puf*M UniR primers (Yutin et al. 2005). The PCR conditions were as follows: initial denaturation for 3 min at 98°C, 30 cycles of 98°C for 10 s, 52°C for 30 s, 72°C for 30 s and final elongation at 72°C for 5 min. PCR was performed in 20 µL triplicate reactions using Phusion™ High-Fidelity PCR MasterMix (ThermoFisher Scientific).

The triplicate reactions for each sample were pooled and purified from the gel using the Wizzard SV Gel and PCR clean system (Promega) and quantified using the Qubit dsDNA HS assay (ThermoFisher Scientific). Amplicons were pooled in equimolar amounts and sequenced on the Illumina MiSeq (2×250 bp) platform at the Genomic Service of the Universitat Pompeu Fabra (Barcelona, Spain).

### Analysis of amplicon data

Reads were quality-checked using FastQC v. 0.11.7 (Babraham Bioinformatics, Cambridge, UK). Primer sequences were trimmed in Cutadapt v. 1.16 (Martin 2011). Subsequent analyses were performed in the R/Bioconductor environment using the DADA2 package v. 1.12.1 (Callahan et al. 2016). *puf*M sequences were processed and assigned using the reference database and methods described in Villena-Alemany et al. (2024). The contamination in the *D. shibae* culture was about 1%. To remove this contamination, the final amplicon sequence variants (ASVs) table consisted of ASVs with the sum of reads in all samples >10 and present in at least two replicates in a treatment at a given time point, or with the sum of reads in all samples >10 and present in at least three time points in a given replicate in a treatment (Piwosz et al. 2018b).

### Statistical analysis

Growth rates were calculated as linear fit coefficients on abundance data transformed with natural logarithm. Differences between incubation in the dark and IR treatment at the end of the experiment were tested with the Welsh t-test. The *P*-values were adjusted for multiple tests using Bonferroni correction, and the significance of the results was assumed for *P* < 0.01. The distribution of the data was tested with the Shapiro–Wilk test. The changes in ASVs’ reads abundance between control and C-limited, and control and lignin/acetate treatments in the IR light at the end of the experiments, were tested using the DESeq function (test=“Wald”, fitType=“parametric”) from DESeq2 package version 1.36.0 (McMurdie and Holmes 2014). The occurrence of specific ASVs between initial and final AAP communities was tested using analysis of compositions of microbiomes with bias correction in ANCOMBC v. 2.3.2 (Lin and Peddada 2020) and plotted using ggplot2 v. 3.4.3. All analyses were performed in Rstudio for Windows (version 2023.03.1+446; R version 4.2.0 (The R Core Team 2021).

### Data availability

The sequences were deposited in the EMBL database under Biosamples ERS17465032-ERS17465210 and ERS17468627 in the BioProject PRJEB71033. The abundance data are available in the PANGAEA information system (Piwosz 2024).

## Results

### June experiment

**Figure 2.**
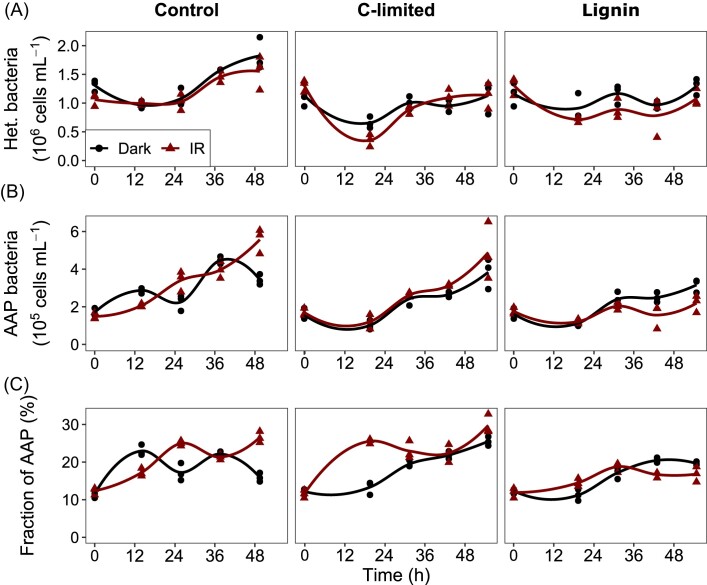
Abundance of heterotrophic bacteria (A); AAP bacteria (B) and contribution of AAP bacteria to total bacterial numbers (C) in the June experiment. Values for each triplicate are shown as points, and the line was fitted locally using the loess function from the ggplot2 package in R.

Heterotrophic bacteria grew fastest in the control treatment, where they almost doubled within 56 h (Fig. 2A, Table 1). The growth rate was slower in the C-limited treatment, while they almost did not grow in the lignin treatment. The growth rate was indifferent between IR light and dark conditions (*P* > 0.01, Table 1).

**Table 1.** Growth rates and abundance of heterotrophic bacteria (Het. bacteria), AAP bacteria (AAP) and contribution of AAP bacteria to total bacterial community (AAP %) at the end of the experiments. *P*-values for statistical significance between IR light and dark conditions are given. Due to multiple testing the significance level was for *P* < 0.01.

			Growth rate (d^−1^)			Abundance (cell mL^−1^) at T_end_		
Exp.	Treatment	Microorganisms	IR light	Dark	*P*-value	IR light	Dark	*P*-value
June	Control	Het. bacteria	0.31 ± 0.03	0.25 ± 0.05	0.57	2.11 ± 0.36×10^6^	2.17 ± 0.34×10^6^	0.58
		AAP	0.66 ± 0.02	0.37 ± 0.07	<0.006	5.57 ± 0.66×10^5^	3.43 ± 0.28×10^5^	<0.009
		AAP %	*NA*			26.6 ± 1.5%	15.9 ± 1.2%	<0.0005
	C-limited	Het. bacteria	0.26 ± 0.13	0.16 ± 0.03	0.37	1.62 ± 0.37×10^6^	1.52 ± 0.36×10^6^	0.37
		AAP	0.55 ± 0.06	0.55 ± 0.04	0.56	4.07 ± 0.78×10^5^	3.84 ± 0.81×10^5^	>0.35
		AAP %	*NA*			29.8 ± 2.6%	25.5 ± 1.2%	<0.043
	Lignin	Het. bacteria	−0.05 ± 0.06	0.12 ± 0.02	0.016	1.30 ± 0.18×10^6^	1.61 ± 0.15×10^6^	>0.05
		AAP	0.12 ± 0.09	0.43 ± 0.02	0.01	2.19 ± 0.45×10^5^	3.16 ± 0.36×10^5^	>0.02
		AAP %	*NA*			16.8 ± 2.0%	19.6 ± 0.5%	0.13
October	Control	Het. bacteria	0.12 ± 0.04	0.06 ± 0.03	0.06	1.59 ± 0.27×10^6^	1.35 ± 0.09×10^6^	0.13
		AAP	0.04 ± 0.05	−0.01 ± 0.03	0.11	0.52 ± 0.08×10^5^	0.62 ± 0.02×10^5^	0.96
		AAP %	*NA*			3.3 ± 0.1%	4.8 ± 0.2%	>0.99
	C-limited	Het. bacteria	0.16 ± 0.02	0.17 ± 0.01	0.71	2.20 ± 0.15×10^6^	1.94 ± 0.12×10^6^	0.04
		AAP	0.46 ± 0.05	0.42 ± 0.07	0.22	3.65 ± 0.15×10^5^	3.09 ± 0.52×10^5^	0.21
		AAP %	*NA*			16.7 ± 1.2%	15.9 ± 1.5%	0.02
	Acetate	Het. bacteria	−0.04 ± 0.05	0.16 ± 0.01	0.01	1.30 ± 0.15×10^6^	2.29 ± 0.36×10^6^	0.01
		AAP	−0.37 ± 0.02	0.15 ± 0.10	0.006	0.58 ± 0.23×10^5^	1.13 ± 0.23×10^5^	0.02
		AAP %	*NA*			4.3 ± 1.1%	5.0 ± 0.9%	0.29

The effect of IR light on AAP bacteria was evident in the control treatment: their growth rate was almost twice as fast in the IR light than in the dark (Table 1). This resulted in a higher abundance and contribution of AAP bacteria in the IR light at the end of the experiment (Fig. 2B and C). Steady growth of AAP bacteria was also observed in the C-limited treatment in both dark and IR light conditions (Fig. 2B and C), but the difference in growth rate, abundance and contribution was insignificant (Table 1). The growth of AAP bacteria did not significantly differ between both dark and IR light conditions in the lignin treatment, which resulted in similar abundances and contributions.

Interestingly, the growth of AAP bacteria in the IR light was significantly slower in the lignin treatment compared with the control (*P* = 0.003), resulting in their lower abundances (*P* = 0.0015) and contribution (*P* = 0.0017) at the end of the experiment. By contrast, the differences in growth rate, abundance and contribution of AAP bacteria between the control and C-limited treatments in the IR light were insignificant. Nevertheless, AAP bacteria grew more than twice as fast as heterotrophic bacteria in the dark and IR light in all treatments (Table 1), indicating that they profited under all of these conditions.

**Figure 3.**
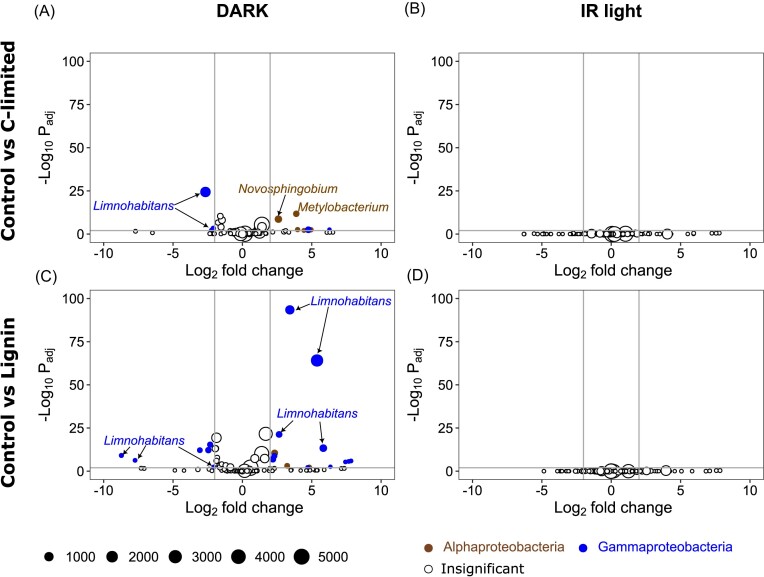
Volcano plots showing the ASVs with significantly different (adjusted *P*-value < 0.01, Log2 fold change > |2|) relative abundance at the end of the experiment between (A and B) control and C-limited treatments, and (C and D) control and lignin treatments in dark (A and C) and IR light (B and D) in the June experiment. A negative Log2 fold change value (x axes) indicates that the read count of an ASV was lower in the experimental treatment than in the control, and a positive value indicates that it was higher. Vertical gray lines show Log2 fold change values of -2 and 2, horizontal gray lines show significance level (adjusted *P*-value < 0.01). Bubble size corresponds to the mean number of reads in both compared treatments, colors show the class affiliation for significant ASVs (brown—Alphaproteobacteria, blue—Gammaproteobacteria, white—insignificant).

The changes in AAP community composition were minor and occurred only in the dark conditions (Supplementary Fig. 1). Only several ASVs significantly altered their relative abundance in C-limited and lignin treatments compared with the control at the end of the experiment in the dark, but not in the IR light (Fig 3). C-limitation induced a relative increase of *Novosphingobium* and *Methylobacterium* (Alphaproteobacteria), and *Limnohabitans* (Gammaproteobacteria) compared with the control treatment. Members of the genus *Limnohabitans* were also affected by lignin treatment, with different ASVs either profiting or losing in these conditions (Fig. 3C). The lack of significant changes in composition between the original and final AAP community in the control (Supplementary Fig. 1) indicates that the growth was induced homogenously within all AAP community.

### October experiment

**Figure 4.**
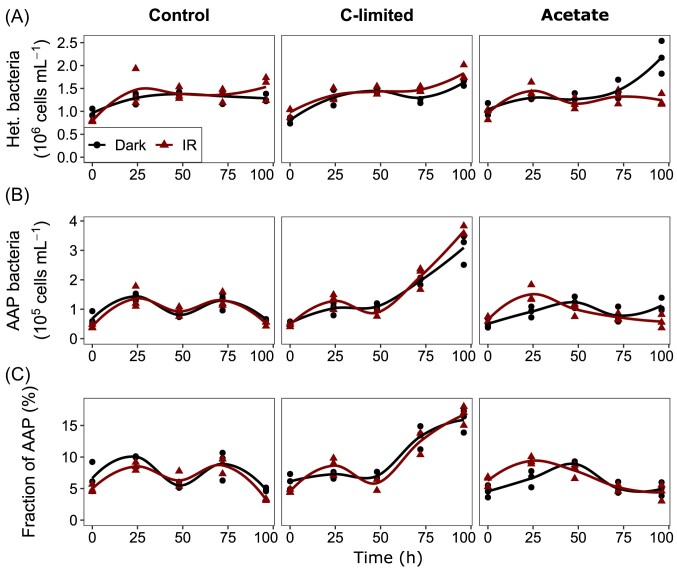
Abundance of heterotrophic bacteria (A); AAP bacteria (B) and contribution of AAP bacteria to total bacterial numbers (C) in the October experiment. Values for each triplicate are shown as points, and the line was fitted locally using loess from the ggplot2 package in R.

The growth patterns in the October experiment were different than in June. Heterotrophic bacteria grew fastest in the C-limited treatment regardless of the light conditions (Table 1), reaching similar abundance at the end of the experiment (Fig. 4A). They grew slower in the control treatment, and the growth rate was similar in the IR light as in the dark, and so was the abundance at the end of the experiment. By contrast, in the acetate treatment, heterotrophic bacteria grew only in the dark, reaching higher abundances at the end of the experiment (Fig. 4A). However, these differences were just at the threshold for statistical significance (Table 1).

The growth rate of AAP bacteria was close to 0 in the control treatment, both in the IR light and dark, and their abundance and contribution to total bacterial abundance did not change (Fig. 4B and C). By contrast, they grew rapidly in the C-limited treatment in both dark and light conditions (Fig. 4B) and their contribution to the total bacterial abundance tripled (Fig. 4C). Finally, in the acetate treatment, AAP bacteria grew in the dark but decreased in the IR light (Table 1), resulting in twice lower abundance in the IR light at the end of the experiment. However, although the growth rate was significantly lower in the acetate than in the control (*P* = 0.002), their abundance and contribution at the end of the experiment did not differ between these treatments (*P* > 0.6). By contrast, AAP bacteria growth rate, final abundance and contribution were significantly higher in the C-limited treatment than in the control (*P* < 0.003).

The growth rate of heterotrophic bacteria compared with AAP bacteria in the IR light did not differ in the control treatment (*P* = 0.94), was significantly lower in the C-limited treatment in IR light (*P* = 0.0023) and was significantly higher in the IR light in the acetate treatment (*P* = 0.003).

Several ASVs significantly changed their relative abundance during the experiment both in the dark and IR light (Supplementary Fig. 1). In the control treatment, *Limnohabitans, Sandarakinorhabdus* and *Hydrogenophaga* increased, while Methylobacteriaceae and Gemmatimonadaceae decreased, especially in the IR treatment. In the C-limited and acetate treatments, the ASVs that increased were affiliated mainly with *Hydrogenophaga*, while those that decreased included Methylobacteriaceae, Gemmatimonadaceae, Pseudomonadales UBA5518 and other Burkholderiaceae (Supplementary Fig. 1).

**Figure 5.**
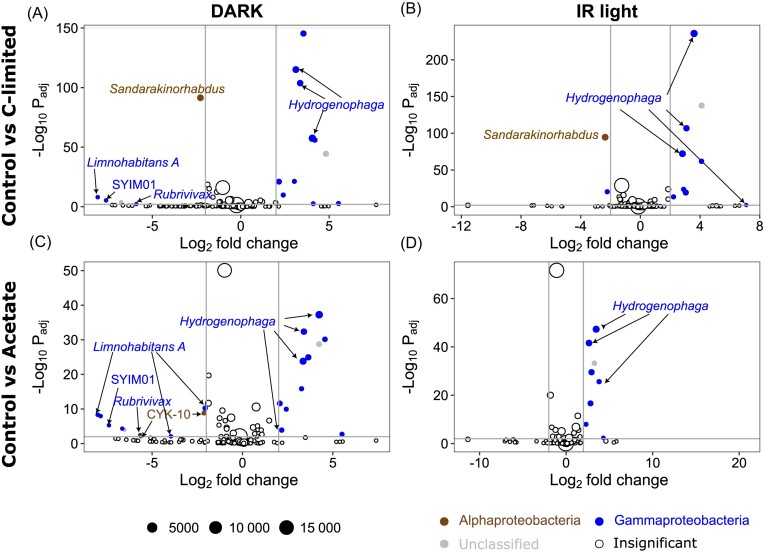
Volcano plots showing the ASV with significantly different (adjusted *P*-value < 0.01, Log2 fold change > |2|) relative abundance at the end of the experiments between (A and B) control and C-limited treatments, and (C and D) control and acetate treatments in dark (A and C) and IR light (B and D) in the October experiment. A negative Log2 fold change value (x axes) indicates that the read count of an ASV was lower in the experimental treatment than in the control, and a positive value indicates that it was higher. Vertical gray lines show Log2 fold change values of −2 and 2, horizontal gray lines show significance level (adjusted *P*-value < 0.01). Bubble size corresponds to the mean number of reads for both compared treatments, colors show the class affiliation for significant ASVs (brown—Alphaproteobacteria, blue—Gammaproteobacteria, grey—unclassified, white—insignificant).

The number of ASVs that showed significantly different relative abundance at the end of the experiment between the control and C-limited or acetate treatments was lower than that observed within the treatments between T0 and Tend. *Hydrogenophaga* increased in the C-limited and acetate treatments compared with the control both in the dark and IR light, while *Limnohabitans* and *Sandarakinorhabdus* decreased, but only in the dark (Fig. 5).

## Discussion

When AAP bacteria were discovered to be abundant in the Northeast Pacific, it was assumed that their ability to use light to produce ATP to support their heterotrophic metabolism helps them to survive in the oligotrophic environment of the ocean (Kolber et al. 2001). However, experimental support for these statements comes mostly from experiments with cultured species using very carbon-rich media (Kopejtka et al. 2020, Kuzyk et al. 2023). By contrast, observations of the distribution and phenology of AAP bacteria in natural environments indicated otherwise: higher concentrations and growth rates were observed in more eutrophic coastal waters during or shortly after the phytoplankton bloom (Koblížek et al. 2007, Auladell et al. 2019, Vrdoljak Tomaš et al. 2019). A similar pattern emerged from freshwater studies (Kolářová et al. 2019, Villena-Alemany et al. 2024), questioning the initial assumption. It was also suggested that additional energy from light can facilitate access to recalcitrant complex organic polymers or low-energy carbon sources, such as lignin or acetate (Koblížek 2015). The question of how AAPs utilize their photoheterotrophy in natural environments remains open.

The results of this study suggest that additional energy from light allows AAP bacteria to successfully compete with heterotrophic bacteria at the time of surplus DOC availability, contradicting the initial hypotheses (Kolber et al. 2001). The support for this statement comes especially from the June experiment, when the growth rate of AAP bacteria in the IR light was significantly higher in the control treatment than in the C-limited treatment (Fig. 2). On the other hand, the negligible growth of AAP bacteria in the control treatment in the October experiment (Fig. 4) indicates that this may be the case only in some seasons or specific environmental conditions. For instance, AAP bacteria seem to be less active and abundant in autumn than in summer (Kolářová et al. 2019, Piwosz et al. 2022, Villena-Alemany et al. 2023). Moreover, there are strong seasonal patterns in AAP community composition in aquatic environments (Villena-Alemany et al. 2024), so the reason for the difference in response between the two experiments could be the higher diversity of AAP bacteria in October (Supplementary Fig. 2). However, more experiments from different seasons and lakes are needed to elucidate how common this effect is.

The growth of AAP bacteria in the C-limited treatment was similar in the IR light and the dark in both seasons (Figs 2 and 4), indicating that they did not profit from photoheterotrophy in such conditions. However, this was contradicted by the observation that they grew much faster than heterotrophic bacteria both in the dark and IR light, increasing their contribution to the total bacterial community up to 3-fold. This incongruence may be explained by several non-exclusive phenomena. For instance, the increase in the fraction of AAP bacteria may have resulted, not from the actual production of new cells by cell division, but from the activation of the photosynthetic genes under carbon limitation in hitherto non-photosynthetic cells (Kopejtka et al. 2020, Kuzyk et al. 2023). On the other hand, AAP bacteria have a quicker metabolism than other heterotrophs (Cepáková et al. 2016, Garcia-Chaves et al. 2016), and the C-limited conditions combined with other features of AAP bacteria may have supported their better growth independently of photoheterotrophy. It may also be possible that although light did not appear to improve the growth of AAP relative to dark conditions, it may have substituted a portion of their respiration requirements.

An unexpected result was the slower growth of the AAP bacteria in the IR light in the lignin treatment in June and the acetate treatment in October compared with the control (Figs 2 and 4). This indicates that both complex polymers (lignin) and low-energy monomers (acetate) may be disadvantageous for freshwater AAP bacteria compared with more bioavailable organic compounds (Steinberg et al. 2006, Hauruseu and Koblížek 2012). This negative relationship, for which the mechanism remains yet unknown, may have serious consequences for lake functioning. Currently, many temperate lakes in the Northern Hemisphere are affected by browning, resulting from the increase in terrestrial dissolved organic matter (DOM) (Williamson et al. 2015). Browning is predicted to continue as the atmospheric acid deposition decreases and due to climate change (Meyer-Jacob et al. 2019), affecting pelagic food webs (Williamson et al. 2015) and increasing CO_2_ flux to the atmosphere (Kritzberg et al. 2020). While some AAP bacteria, such as *Sphingomonas* sp. strain FukuSWIS1 from the acidic lake Grosse Fuchskuhle (Salka et al. 2014), seem to be adapted to conditions prevailing in humic and brown lakes, our results indicate that overall AAP bacteria may be negatively impacted by recalcitrant or low energy carbon sources. This effect may be more pronounced in wet seasons, such as spring and autumn, when terrestrial DOM inputs, potentially higher due to river runoff and falling leaves, could hamper their photoheterotrophy, decreasing the efficiency of carbon assimilation and lowering its availability for higher trophic levels (Piwosz et al. 2022, Villena-Alemany et al. 2024). Further experiments employing a wider variety of recalcitrant and low-energy compounds are needed to confirm and understand this effect.

AAP community composition shows recurrent seasonal patterns in freshwater lakes (Villena-Alemany et al. 2024), which are driven by changes in environmental conditions, such as temperature and DOC concentration (Villena-Alemany et al. 2023). The minor changes in the AAP community composition under IR light observed here indicate that the response to the treatments could have been driven by a physiological change in specific AAP phylotypes switching to phototrophic metabolisms rather than a community-level response and production of new cells of AAP bacteria (Fecskeová et al. 2019, Piwosz et al. 2020). For instance, the only genus that had significantly increased its relative abundance in IR light was *Hydrogenophaga* in October (Fig. 5B and D). Members of this genus were reported to oxidize hydrogen as an energy source (Willems et al. 1989), which may have interesting implications for the functional role of AAP bacteria in freshwaters. In addition, numerous ASVs affiliated with *Limnohabitans* either increased or decreased in lignin treatment in June (Fig. 3C), which may indicate niche separation between closely related AAP species (Villena-Alemany et al. 2024).

It is also important to notice that none of the typical anaerobic purple non-sulphur bacteria (PNSB) phylotypes, such as *Rubrivivax* or *Rhodoferax*, were present in larger numbers (Supplementary Figs 1 and 2), supporting the attribution of observed responses to the activity of AAP bacteria.

Interestingly, more ASVs changed their relative abundance under dark conditions throughout the experiment, especially in June (Figs 3 and 5, Supplementary Fig. 1). This suggests that dark incubations, which are commonly used in experimental design to minimize the effect of primary producers (Šimek et al. 2020, Fecskeová et al. 2021), may exaggerate the community-level responses compared with light treatments (Piwosz et al. 2020). This observation aids in arguments that dark incubations provide biased insights into the activity of freshwater bacterioplankton (Piwosz et al. 2022).

## Conclusions

Our experimental evidence indicates that although AAP bacteria's ability to use light as a supplementary energy source is induced under carbon limitation, they can also profit from photoheterotrophy when carbon is available. However, their advantage over heterotrophic bacteria may depend on the specific environmental conditions. This effect also seems to be driven by physiological responses rather than changes at the community level. These findings contribute to our understanding of the ecological role of AAP bacteria in lakes. Finally, the observation of the negative effect of lignin and acetate on AAP bacteria opens a new research topic in their ecology, because it may have ecosystem-level consequences as lake browning continues.

## Supplementary Material

fiae090_Supplemental_File

## References

[bib1] Auladell A, Sánchez P, Sánchez O et al. Long-term seasonal and interannual variability of marine aerobic anoxygenic photoheterotrophic bacteria. ISME J. 2019;13:1975–87. 10.1038/s41396-019-0401-4. 30914777 PMC6776013

[bib2] Bugg TDH, Ahmad M, Hardiman EM et al. Pathways for degradation of lignin in bacteria and fungi. Nat Prod Rep. 2011;28:1883–96. 10.1039/C1NP00042J. 21918777

[bib3] Callahan BJ, McMurdie PJ, Rosen MJ et al. DADA2: high-resolution sample inference from Illumina amplicon data. Nat Methods. 2016;13:581–3. 10.1038/nmeth.3869. 27214047 PMC4927377

[bib4] Cepáková Z, Hrouzek P, Žišková E et al. High turnover rates of aerobic anoxygenic phototrophs in European freshwater lakes. Environ Microbiol. 2016;18:5063–71. 10.1111/1462-2920.13475. 27485742

[bib5] Coleman AW . Enhanced detection of bacteria in natural environments by fluorochrome staining of DNA. Limnol Oceanogr. 1980;25:948–51. 10.4319/lo.1980.25.5.0948.

[bib6] Cottrell MT, Mannino A, Kirchman DL. Aerobic anoxygenic phototrophic bacteria in the Mid-Atlantic Bight and the North Pacific Gyre. Appl Environ Microb. 2006;72:557–64. 10.1128/AEM.72.1.557-564.2006. PMC1352302 16391092

[bib7] Čuperová Z, Holzer E, Salka I et al. Temporal changes and altitudinal distribution of aerobic anoxygenic phototrophs in mountain lakes. Appl Environ Microb. 2013;79:6439–46. 10.1128/aem.01526-13. PMC3811222 23956384

[bib8] Fauteux L, Cottrell MT, Kirchman DL et al. Patterns in abundance, cell size and pigment content of aerobic anoxygenic phototrophic bacteria along environmental gradients in northern lakes. PLoS One. 2015;10:e0124035. 10.1371/journal.pone.0124035. 25927833 PMC4415779

[bib9] Fecskeová LK, Piwosz K, Hanusová M et al. Diel changes and diversity of *puf*M expression in freshwater communities of anoxygenic phototrophic bacteria. Sci Rep. 2019;9:18766. 10.1038/s41598-019-55210-x. 31822744 PMC6904477

[bib10] Fecskeová LK, Piwosz K, Šantić D et al. Lineage-specific growth curves document large differences in response of individual groups of marine bacteria to the top-down and bottom-up controls. Msystems. 2021;6:e00934–00921. 10.1128/mSystems.00934-21. PMC8547455 34581594

[bib11] Garcia-Chaves MC, Cottrell MT, Kirchman DL et al. Single-cell activity of freshwater aerobic anoxygenic phototrophic bacteria and their contribution to biomass production. ISME J. 2016;10:1579–88. 10.1038/ismej.2015.242. 26771928 PMC4918449

[bib12] Gómez-Consarnau L, Raven JA, Levine NM et al. Microbial rhodopsins are major contributors to the solar energy captured in the sea. Sci Adv. 2019;5:eaaw8855. 10.1126/sciadv.aaw8855. 31457093 PMC6685716

[bib13] Griffiths RI, Whiteley AS, O'Donnell AG et al. Rapid method for coextraction of DNA and RNA from natural environments for analysis of ribosomal DNA- and rRNA-based microbial community composition. Appl Environ Microb. 2000;66:5488–91. 10.1128/aem.66.12.5488-5491.2000. PMC92488 11097934

[bib14] Hahn MW, Lunsdorf H, Wu QL et al. Isolation of novel ultramicrobacteria classified as Actinobacteria from five freshwater habitats in Europe and Asia. Appl Environ Microb. 2003;69:1442–51. 10.1128/aem.69.3.1442-1451.2003. PMC150105 12620827

[bib15] Hauruseu D, Koblížek M. Influence of light on carbon utilization in aerobic anoxygenic phototrophs. Appl Environ Microb. 2012;78:7414–9. 10.1128/aem.01747-12. PMC3457121 22885759

[bib16] Kasalický V, Zeng Y, Piwosz K et al. Aerobic anoxygenic photosynthesis is commonly present within the genus *Limnohabitans*. Appl Environ Microb. 2018;84:e02116–02117. 10.1128/aem.02116-17. PMC5734016 29030444

[bib18] Koblížek M, Dachev M, Bína D et al. Utilization of light energy in phototrophic Gemmatimonadetes. J Photochem Photobiol B: Biol. 2020;213:112085. 10.1016/j.jphotobiol.2020.112085. 33220599

[bib19] Koblížek M, Masin M, Ras J et al. Rapid growth rates of aerobic anoxygenic phototrophs in the ocean. Environ Microbiol. 2007;9:2401–6. 10.1111/j.1462-2920.2007.01354.x. 17803766

[bib20] Koblížek M, Mlčoušková J, Kolber Z et al. On the photosynthetic properties of marine bacterium COL2P belonging to *Roseobacter* clade. Arch Microbiol. 2010;192:41–9. 10.1007/s00203-009-0529-0. 19949940

[bib17] Koblížek M . Ecology of aerobic anoxygenic phototrophs in aquatic environments. FEMS Microbiol Rev. 2015;39:854–70. 10.1093/femsre/fuv032. 26139241

[bib21] Kolářová E, Medová H, Piwosz K et al. Seasonal dynamics of aerobic anoxygenic phototrophs in freshwater lake Vlkov. Folia Microbiol. 2019;64:705–10. 10.1007/s12223-019-00735-x. 31346963

[bib22] Kolber ZS, Plumley FG, Lang AS et al. Contribution of aerobic photoheterotrophic bacteria to the carbon cycle in the ocean. Science. 2001;292:2492–5. 10.1126/science.1059707. 11431568

[bib23] Kopejtka K, Tomasch J, Zeng Y et al. Simultaneous presence of Bacteriochlorophyll and Xanthorhodopsin genes in a freshwater bacterium. Msystems. 2020;5:e01044–01020. 10.1128/mSystems.01044-20. 33361324 PMC7762795

[bib24] Kopejtka K, Zeng Y, Kaftan D et al. Characterization of the aerobic anoxygenic phototrophic bacterium *Sphingomonas* sp. AAP5. Microorganisms. 2021;9:768. 10.3390/microorganisms9040768. 33917603 PMC8067484

[bib25] Kritzberg ES, Hasselquist EM, Škerlep M et al. Browning of freshwaters: consequences to ecosystem services, underlying drivers, and potential mitigation measures. Ambio. 2020;49:375–90. 10.1007/s13280-019-01227-5. 31367885 PMC6965042

[bib26] Kuzyk SB, Messner K, Plouffe J et al. Diverse aerobic anoxygenic phototrophs synthesize bacteriochlorophyll in oligotrophic rather than copiotrophic conditions, suggesting ecological niche. Environ Microbiol. 2023;25:2653–65. 10.1111/1462-2920.16482. 37604501

[bib27] Lew S, Koblížek M, Lew M et al. Seasonal changes of microbial communities in two shallow peat bog lakes. Folia Microbiol. 2015;60:165–75. 10.1007/s12223-014-0352-0. 25331011

[bib28] Lin H, Peddada SD. Analysis of compositions of microbiomes with bias correction. Nat Commun. 2020;11:3514. 10.1038/s41467-020-17041-7. 32665548 PMC7360769

[bib29] Martin M . Cutadapt removes adapter sequences from high-throughput sequencing reads. EMBnet Journal. 2011;17:10–2. 10.14806/ej.17.1.200.

[bib30] Masin M, Cuperova Z, Hojerova E et al. Distribution of aerobic anoxygenic phototrophic bacteria in glacial lakes of northern Europe. Aquat Microb Ecol. 2012;66:77–86. 10.3354/ame01558.

[bib31] Masin M, Nedoma J, Pechar L et al. Distribution of aerobic anoxygenic phototrophs in temperate freshwater systems. Environ Microbiol. 2008;10:1988–96. 10.1111/j.1462-2920.2008.01615.x. 18430010

[bib32] McMurdie PJ, Holmes S. Waste not, want not: why rarefying microbiome data is inadmissible. PLoS Comp Biol. 2014;10:e1003531. 10.1371/journal.pcbi.1003531. PMC3974642 24699258

[bib33] Meyer-Jacob C, Michelutti N, Paterson AM et al. The browning and re-browning of lakes: divergent lake-water organic carbon trends linked to acid deposition and climate change. Sci Rep. 2019;9:16676. 10.1038/s41598-019-52912-0. 31723150 PMC6853936

[bib34] Nercessian O, Noyes E, Kalyuzhnaya MG et al. Bacterial populations active in metabolism of C-1 compounds in the sediment of Lake Washington, a freshwater lake. Appl Environ Microb. 2005;71:6885–99. 10.1128/aem.71.11.6885-6899.2005. PMC1287692 16269723

[bib35] Okamura K, Mitsumori F, Ito O et al. Photophosphorylation and oxidative phosphorylation in intact cells and chromatophores of an aerobic photosynthetic bacterium, *Erythrobacter* sp. strain OCh114. J Bacteriol. 1986;168:1142–6. 10.1128/jb.168.3.1142-1146.1986. 3782035 PMC213614

[bib37] Piwosz K, Kaftan D, Dean J et al. Non-linear effect of irradiance on photoheterotrophic activity and growth of the aerobic anoxygenic phototrophic bacterium *Dinoroseobacter shibae*. Environ Microbiol. 2018a;20:724–33. 10.1111/1462-2920.14003. 29159858

[bib36] Piwosz K, Całkiewicz J, Gołębiewski M et al. Diversity and community composition of pico- and nanoplanktonic protists in the Vistula River estuary (Gulf of Gdańsk, Baltic Sea). Estuar Coast Shelf Sci. 2018b;207:242–9. 10.1016/j.ecss.2018.04.013.

[bib38] Piwosz K, Villena-Alemany C, Mujakić I. Photoheterotrophy by aerobic anoxygenic bacteria modulates carbon fluxes in a freshwater lake. ISME J. 2022;16:1046–54. 10.1038/s41396-021-01142-2. 34802055 PMC8941148

[bib39] Piwosz K, Vrdoljak A, Frenken T et al. Light and primary production shape bacterial activity and community composition of aerobic anoxygenic phototrophic bacteria in a microcosm experiment. mSphere. 2020;5:e00354–00320. 10.1128/mSphere.00354-20. 32611696 PMC7333569

[bib40] Piwosz K . Response of aerobic anoxygenic phototrophic bacteria to carbon limitation. Dataset, PANGAEA, 2024. 10.1594/PANGAEA.967435. PMC11229431 38886127

[bib41] Ruiz-González C, Garcia-Chaves MC, Ferrera I et al. Taxonomic differences shape the responses of freshwater aerobic anoxygenic phototrophic bacterial communities to light and predation. Mol Ecol. 2020;29:1267–83. 10.1111/mec.15404. 32147876

[bib42] Salka I, Srivastava A, Allgaier M et al. The draft genome sequence of *Sphingomonas* sp. strain FukuSWIS1, obtained from acidic lake Grosse Fuchskuhle, indicates photoheterotrophy and a potential for humic matter degradation. Genome Announc. 2014;2:e01183–01114. 10.1128/genomeA.01183-14. PMC4241673 25395647

[bib43] Shabarova T, Salcher MM, Porcal P et al. Recovery of freshwater microbial communities after extreme rain events is mediated by cyclic succession. Nat Microbiol. 2021;6:479–88. 10.1038/s41564-020-00852-1. 33510474

[bib44] Shiba T, Shioi Y, Takamiya K-I et al. Distribution and physiology of aerobic bacteria containing Bacteriochlorophyll *a* on the east and west coasts of Australia. Appl Environ Microb. 1991;57:295–300. 10.1128/aem.57.1.295-300.1991. PMC182701 16348398

[bib45] Shiba T, Simidu U, Taga N. Distribution of aerobic bacteria which contain Bacteriochlorophyll *a*. Appl Environ Microb. 1979;38:43–5. 10.1128/aem.38.1.43-45.1979. PMC243433 16345414

[bib46] Šimek K, Grujčić V, Mukherjee I et al. Cascading effects in freshwater microbial food webs by predatory Cercozoa, Katablepharidacea and ciliates feeding on aplastidic bacterivorous cryptophytes. FEMS Microbiol Ecol. 2020;96:fiaa121. 10.1093/femsec/fiaa121. 32556274 PMC7538307

[bib47] Steinberg CEW, Kamara S, Prokhotskaya VY et al. Dissolved humic substances—ecological driving forces from the individual to the ecosystem level?. Freshwat Biol. 2006;51:1189–210. 10.1111/j.1365-2427.2006.01571.x.

[bib48] The R Core Team . R: a language and environment for statistical computing. R Foundation for Statistical Computing, Vienna, Austria, 2021. http://www.R-project.org/ (31 March 2024, date last accessed).

[bib49] Villena-Alemany C, Mujakic I, Fecskeova LK et al. Phenology and ecological role of aerobic anoxygenic phototrophs in fresh waters. Microbiome. 2024;12:65. 10.1186/s40168-024-01786-0. 38539229 PMC10976687

[bib50] Villena-Alemany C, Mujakić I, Porcal P et al. Diversity dynamics of aerobic anoxygenic phototrophic bacteria in a freshwater lake. Environ Microbiol Rep. 2023;15:60–71. 10.1111/1758-2229.13131. 36507772 PMC10103773

[bib51] Vrdoljak Tomaš A, Šantić D, Šolić M et al. Dynamics of aerobic anoxygenic phototrophs along the trophic gradient in the central Adriatic Sea. Deep Sea Res Part II. 2019;164:112–21. 10.1016/j.dsr2.2019.06.001.

[bib52] Willems A, Busse J, Goor M et al. *Hydrogenophaga*, a new genus of hydrogen-oxidizing bacteria that includes *Hydrogenophaga flava* comb. nov. (formerly *Pseudomonas flava*), *Hydrogenophaga palleronii* (formerly *Pseudomonas palleronii*), *Hydrogenophaga pseudoflava* (formerly *Pseudomonas pseudoflava* and “*Pseudomonas carboxydoflava*”), and *Hydrogenophaga taeniospiralis* (formerly *Pseudomonas taeniospiralis*). Int J Syst Evol Microbiol. 1989;39:319–33. 10.1099/00207713-39-3-319.

[bib53] Williamson CE, Overholt EP, Pilla RM et al. Ecological consequences of long-term browning in lakes. Sci Rep. 2015;5:18666. 10.1038/srep18666. 26690504 PMC4687041

[bib54] Yurkov V, Gorlenko VM. *Erythrobacter sibiricus* sp. nov., a new freshwater aerobic bacterial species containing bacteriochlorophyll *a*. Microbiology. 1990;59:85–9.

[bib55] Yurkov VV, Beatty JT. Aerobic anoxygenic phototrophic bacteria. Microbiol Mol Biol Rev. 1998;62:695–724. 9729607 10.1128/mmbr.62.3.695-724.1998 PMC98932

[bib56] Yurkov VV, Van Gemerden H. Impact of light/dark regimen on growth rate, biomass formation and bacteriochlorophyll synthesis in *Erythromicrobium hydrolyticum*. Arch Microbiol. 1993;159:84–9. 10.1007/bf00244268.

[bib57] Yutin N, Suzuki MT, Béjà O. Novel primers reveal wider diversity among marine aerobic anoxygenic phototrophs. Appl Environ Microb. 2005;71:8958–62. 10.1128/aem.71.12.8958-8962.2005. PMC1317425 16332899

