# Supplementary material for: Response of aerobic anoxygenic phototrophic bacteria to limitation and availability of organic carbon
Source: FEMS Microbiol Ecol. 2024 Jun 17;100(7):fiae090. doi: 10.1093/femsec/fiae090 (PMC11229431; doi:10.1093/femsec/fiae090)
Supplement: fiae090_Supplemental_File [file fiae090_supplemental_file.pdf]

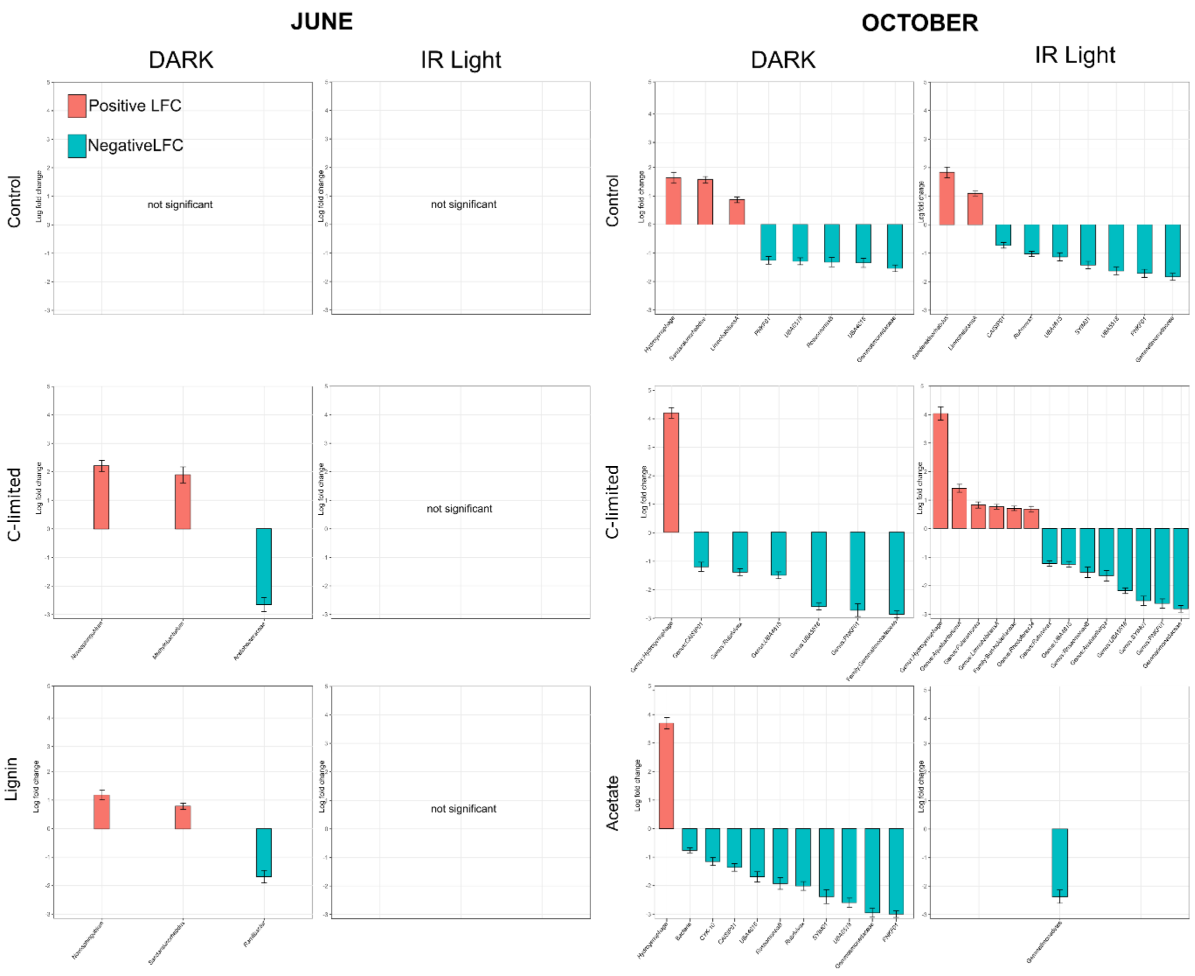

Supplementary Figure 1. Analysis of composition of microbiomes showing log (natural logarithm) fold change (LFC) values at genus level between initial and final communities in the experimental treatments under different light conditions in June and October experiments. Error bars represent standard error.

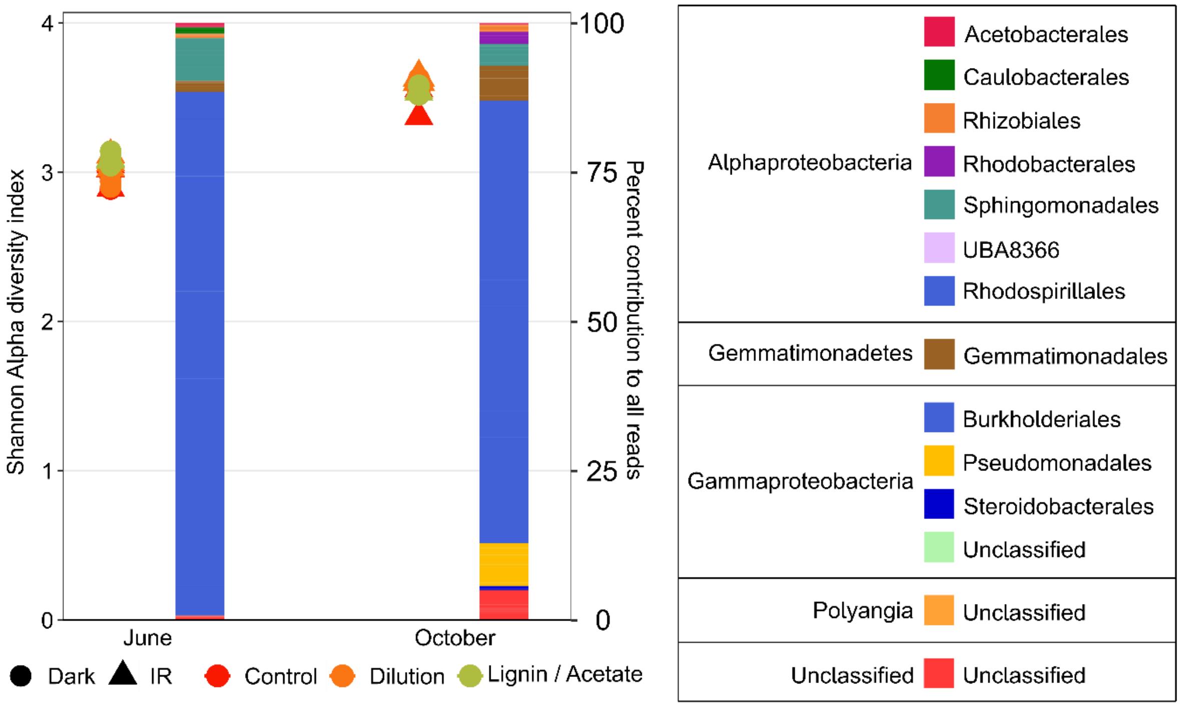

Supplementary Figure 2. Shannon alpha diversity index and community composition of AAP bacteria based on *puf*M gene amplicon at Time 0 in June and October experiments.
